# Roles of iron acquisition systems in virulence of extraintestinal pathogenic *Escherichia coli*: salmochelin and aerobactin contribute more to virulence than heme in a chicken infection model

**DOI:** 10.1186/1471-2180-12-143

**Published:** 2012-07-20

**Authors:** Qingqing Gao, Xiaobo Wang, Huiqing Xu, Yaya Xu, Jielu Ling, Debao Zhang, Song Gao, Xiufan Liu

**Affiliations:** 1 Animal Infectious Disease Laboratory, Ministry of Education Key Lab for Avian Preventive Medicine, College of Veterinary Medicine, Yangzhou University, Yangzhou, Jiangsu, 225009, People's Republic of China

**Keywords:** APEC, Avian pathogenic *Escherichia coli*, UPEC, Uropathogenic *Escherichia coli*, Iron acquisition system, Salmochelin, Aerobactin, Heme, Pathogenicity, Chicken challenge model

## Abstract

**Background:**

Avian pathogenic *Escherichia coli* (APEC) and uropathogenic *E. coli* (UPEC) are the two main subsets of extraintestinal pathogenic *E. coli* (ExPEC). Both types have multiple iron acquisition systems, including heme and siderophores. Although iron transport systems involved in the pathogenesis of APEC or UPEC have been documented individually in corresponding animal models, the contribution of these systems during simultaneous APEC and UPEC infection is not well described. To determine the contribution of each individual iron acquisition system to the virulence of APEC and UPEC, isogenic mutants affecting iron uptake in APEC E058 and UPEC U17 were constructed and compared in a chicken challenge model.

**Results:**

Salmochelin-defective mutants E058**Δ***iroD* and U17**Δ***iroD* showed significantly decreased pathogenicity compared to the wild-type strains. Aerobactin defective mutants E058**Δ***iucD* and U17**Δ***iucD* demonstrated reduced colonization in several internal organs, whereas the heme defective mutants E058**Δ***chuT* and U17**Δ***chuT* colonized internal organs to the same extent as their wild-type strains. The triple mutant **Δ***chuT***Δ***iroD***Δ***iucD* in both E058 and U17 showed decreased pathogenicity compared to each of the single mutants. The histopathological lesions in visceral organs of birds challenged with the wild-type strains were more severe than those from birds challenged with **Δ***iroD*, **Δ***iucD* or the triple mutants. Conversely, chickens inoculated with the **Δ***chuT* mutants had lesions comparable to those in chickens inoculated with the wild-type strains. However, no significant differences were observed between the mutants and the wild-type strains in resistance to serum, cellular invasion and intracellular survival in HD-11, and growth in iron-rich or iron-restricted medium.

**Conclusions:**

Results indicated that APEC and UPEC utilize similar iron acquisition mechanisms in chickens. Both salmochelin and aerobactin systems appeared to be important in APEC and UPEC virulence, while salmochelin contributed more to the virulence. Heme bounded by ChuT in the periplasm appeared to be redundant in this model, indicating that other periplasmic binding proteins likely contributed to the observed no phenotype for the heme uptake mutant. No differences were observed between the mutants and their wild-type parents in other phenotypic traits, suggesting that other virulence mechanisms compensate for the effect of the mutations.

## Background

Extraintestinal pathogenic *Escherichia coli* (ExPEC) refers to a group of strains capable of causing diseases outside the intestinal tract, including uropathogenic *E. coli* (UPEC), sepsis-associated *E. coli*, and neonatal meningitis-associated *E. coli*[1]. Among ExPEC strains, UPEC is the most common cause of human urinary tract infections (UTIs) [2,3]. Avian pathogenic *E. coli* (APEC) is the main cause of avian colibacillosis, which refers to any localized or systemic infections such as acute fatal septicemia or subacute pericarditis and airsacculitis. APEC and UPEC possess similar virulence factors for colonizing and invading the host, including adhesins, toxins, polysaccharide coatings, protectins, invasins, and iron acquisition systems [4,5].

Iron is an essential element for survival of *E. coli*. It facilitates numerous cellular activities, such as peroxide reduction, electron transport, and nucleotide biosynthesis [6-9]. As iron exists at low concentrations in extraintestinal sites of infection, the ExPEC strains have evolved multiple strategies for sequestering iron from the host.

The direct way is to take up iron from either free heme or from heme-containing proteins, such as hemoglobin or hemopexin. Heme is the most abundant iron source *in vivo*, and the presence of a heme system in ExPEC strains may be important for the acquisition of iron from heme or hemoglobin. Specific outer membrane receptors Hma and ChuA bind host hemoproteins and transfer the coordinated heme molecule into the periplasm, where an ABC transport system delivers it to the cytoplasm. Once taken up by ChuA and transported across the outer membrane, heme is internalized into the periplasm and then bound by heme-specific periplasmic transport protein ChuT, which mediates heme transfer to the cytoplasm through an ATP-binding cassette (ABC) transporter [10].

The indirect strategy for iron acquisition is based on a shuttle mechanism, which uses small-molecule compounds called siderophores as high-affinity ferric iron chelators [11], including the catecholates enterobactin, salmochelin, the hydroxamate aerobactin, and yersiniabactin [12]. Salmochelin molecules were first discovered in *Salmonella enterica*[13]. The *iroA* locus responsible for salmochelin production was also first identified in *Salmonella spp*. [14]. Salmochelins are C-glucosylated derivatives of enterobactin, encoded by the *iroBCDEN* gene cluster [15]. Among *E. coli* isolates, *iro* sequences have been described in ExPEC strains isolated from patients with neonatal meningitis [16], UTIs, and prostatitis in humans [17,18], as well as from APEC isolates from poultry. Compared to enterobactins, salmochelins are superior siderophores in the presence of serum albumin, which may suggest that salmochelins are considerably more important in the pathogenesis of certain *E. coli* and *Salmonella* infections than enterobactins [19]. In ExPEC strains, the gene cluster responsible for salmochelin biosynthesis and transport is generally found on ColV or ColBM virulence plasmids, and has also been identified on chromosomal pathogenicity-associated islands (PAI) in some strains [20]. The salmochelin gene cluster contains a gene encoding a cytoplasmic esterase, IroD. IroD can hydrolyze the ester bonds of both enterobactin and salmochelin molecules, which is required for subsequent iron release from salmochelin [21,22].

Aerobactin is a hydroxamate siderophore produced by most APEC strains and other pathogenic *E. coli*. It is synthesized by the *iucABCD*-encoded gene products and taken up by the *iutA*-encoded receptor protein [23-25]. Despite the chemical differences among these distinct siderophores, each system is comprised of components mediating the specific steps required for ferric iron uptake, including siderophore synthesis in the cytoplasm, secretion, reception of the ferri-siderophore at the outer membrane surface, internalization, and iron release in the cytoplasm [26].

While both APEC and UPEC strains have multiple iron acquisition systems, the role of distinct iron uptake systems in the pathogenesis of both APEC and UPEC has not been illustrated in the same chicken challenge model. In this study, the genes *chuT*, *iroD* and *iucD* were chosen to assert the roles of heme, salmochelin and aerobactin in the virulence of APEC E058 and UPEC U17.

## Results

### Iron acquisition systems in APEC E058 and UPEC U17

APEC E058 belongs to the O2 serogroup and B2 phylogenetic group, while UPEC U17, isolated from the urine of a patient, was found to belong to the B2 phylogenetic group, but was non-typable by the standard O sera test. Previous work in our laboratory has shown that E058 and U17 share similar virulence gene profiles and that both cause a typical avian colibacillosis, with bacteria invading the air sacs, blood, and pericardial fluid, with typical fibrinous lesions. Both strains possess the same iron uptake systems, including heme, enterobactin, salmochelin, aerobactin, and yersiniabactin [5].

### Effect of iron acquisition system mutations on chicken virulence

**Figure 1:**
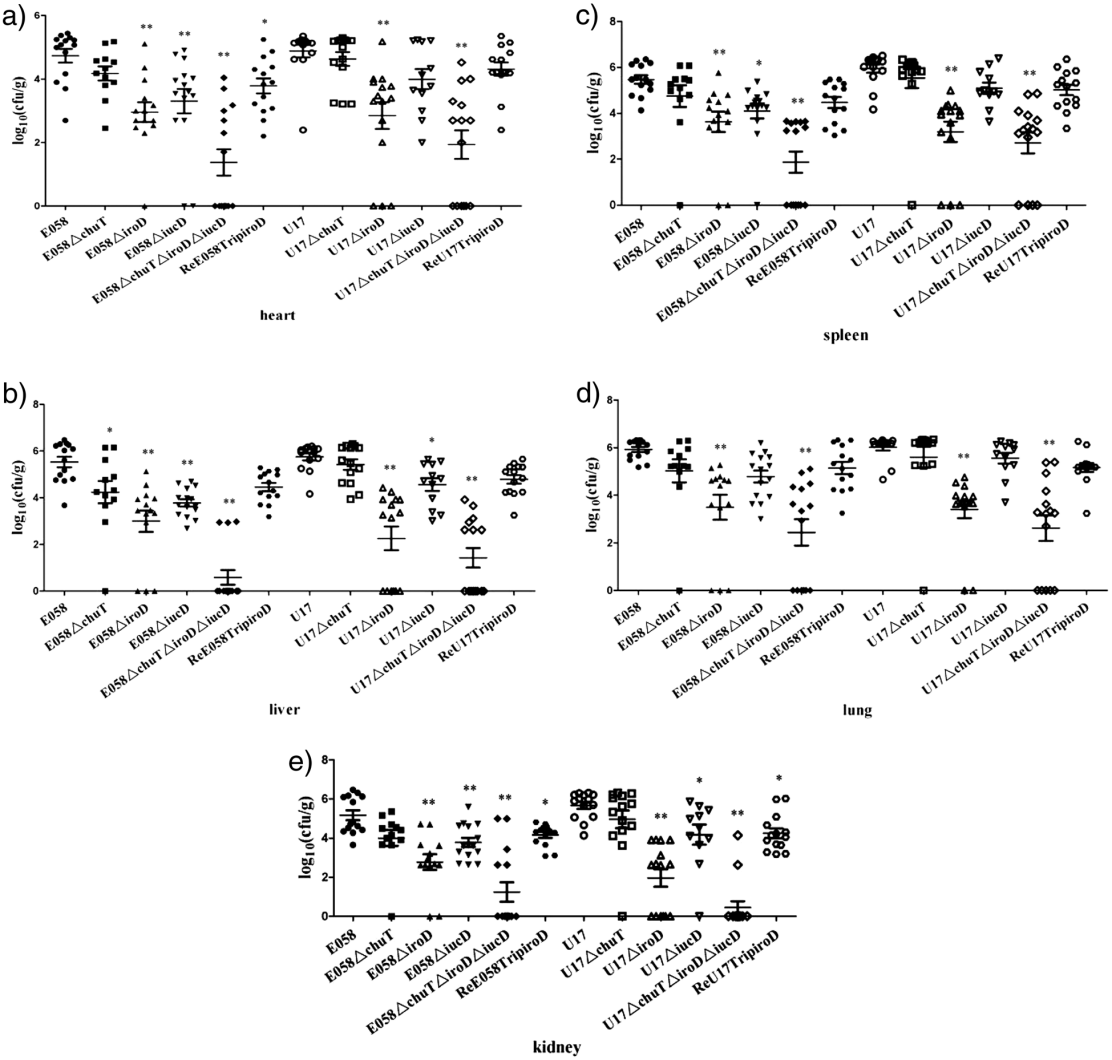
**Colonization in organs of chickens challenged with APEC E058, UPEC U17, or their isogenic mutants in the single-strain challenge model.** Data are presented as log_10_(CFU/g) of tissues. Horizontal bars indicate the mean log_10_ CFU.g^-1^ values. Each data point represents a tissue sample from an individual infected chicken at 24h post-infection. Organs sampled were the heart (**a**), liver (**b**), spleen (**c**), lung (**d**), and kidney (**e**). Statistically significant decreases in bacterial loads are indicated with asterisks (*, P<0.05; * *, P<0.01).

Because iron acquisition systems were associated with *E. coli* isolates from extraintestinal infections, we investigated the importance of distinct iron uptake systems to the virulence of APEC E058 and UPEC U17 in chickens. In the single-strain challenge model, 5-week-old chickens were inoculated in the left thoracic air sac with wild-type strains or their isogenic mutant derivatives. From the inoculation site, virulent strains can typically invade deeper tissues, generate gross lesions, and cause systemic infection. However, in this model, attenuated strains are impaired in their capacity to colonize deeper tissues. Compared to wild-type parent strains, both the mutants E058Δ*iroD* and U17Δ*iroD* were attenuated, and significantly reduced bacterial numbers were recovered from all internal organs tested: 10–100 times lower than those of the wild-type strains (P<0.01) (Figure 1). E058Δ*iucD* showed significantly reduced bacterial numbers in the heart (Figure 1a), liver (Figure 1b), kidney (Figure 1e) (P<0.01), and spleen (Figure 1c) (P<0.05). Meanwhile, U17Δ*iucD* had significantly decreased bacteria counts in both the liver (Figure 1b) and kidney (Figure 1e) (P<0.05). The E058Δ*chuT* and U17Δ*chuT* colony forming units (CFU) isolated from the organs of the chickens were similar to those of the wild-type strains (Figure 1) (P>0.05), except for E058Δ*chuT* in liver tissue (Figure 1b) (P<0.05). Challenge with the E058Δ*chuT*Δ*iroD*Δ*iucD* and U17Δ*chuT*Δ*iroD*Δ*iucD* triple mutants led to greatest reductions in bacterial loads in all the tested internal organs (Figure 1) (P<0.01). To determine whether the defect in the triple mutants was mainly mediated by the salmochelin system, we constructed a complementation plasmid for the triple mutants using the native *iroD* gene. Results showed that the recovered colony numbers of ReE058Trip*iroD* isolated from organs were similar to those of the wild-type strain in liver (Figure 1b), spleen (Figure 1c), lung (Figure 1d) (P>0.05). Meanwhile, the recovered CFU of ReU17Trip*iroD* in heart (Figure 1a), liver (Figure 1b), spleen (Figure 1c), and lung (Figure 1d) were similar to those of the wild-type strain (P>0.05).

**Figure 2:**
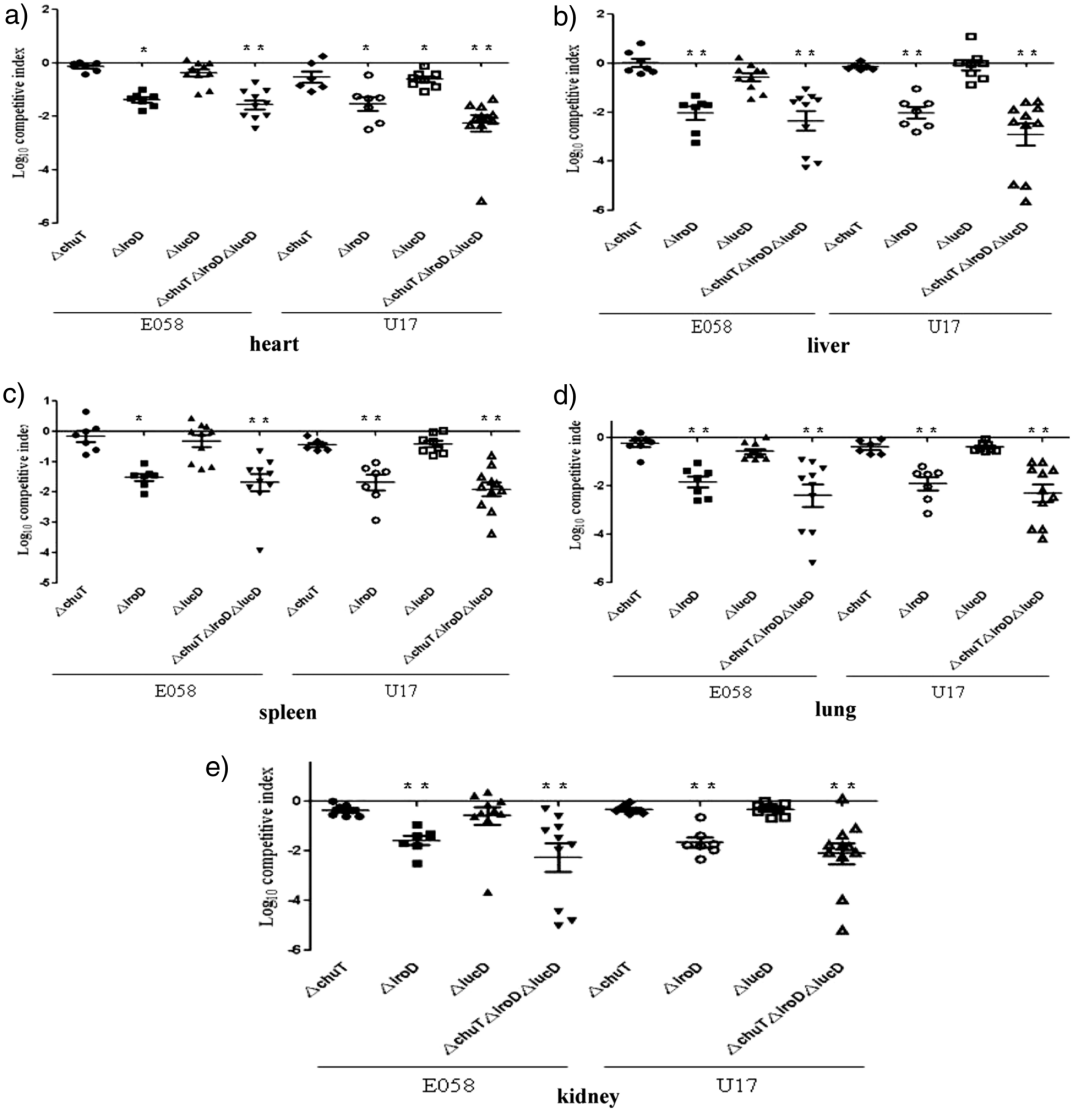
**Competitive co-infection model was used in which E058 or U17 and isogenic mutants were inoculated simultaneously.** At 24 h post-infection, tissues were sampled, and results are presented as the log_10_ competitive index (CI). The CI represents the relative numbers of the two test strains from the tissues sampled (the output ratio) compared to the initial numbers of the strains in the inoculum (input ratio). Negative CI values indicate a decreased capacity for the mutant to compete with the virulent wild-type strain. Horizontal bars indicate the mean log_10_ CI values. Organs sampled were the heart (**a**), liver (**b**), spleen (**c**), lung (**d**), and kidney (**e**). Statistically significant decreases in CI values are indicated with asterisks (*, p<0.05; **, p<0.01).

Compared to the single-strain challenge model, the competitive co-infection model using both parent strain and its isogenic mutant can demonstrate more sensitivity to differences in colonization or virulence. In co-infection experiments, both E058Δ*chuT* and E058Δ*iucD* did not demonstrate any significant decrease in pathogenicity compared to E058 wild-type in organs (Figure 2) (P>0.05), while E058Δ*iroD* was highly attenuated and showed a significantly reduced competitive index (CI), with mean decreases of 77–fold, 70-fold, and 37–fold compared to E058 in liver (Figure 2b), lung (Figure 2d) and kidney (Figure 2e) (P<0.01), respectively. For U17 and its isogenic mutants, U17Δ*chuT* demonstrated no significant decreases compared to U17 in all internal organs tested (Figure 2) (P>0.05), while U17Δ*iroD* CFU counts were highly reduced, with mean decreases of 105-fold, 49-fold, 80-fold, and 46-fold compared to the wild-type strain in liver (Figure 2b), spleen (Figure 2c), lung (Figure 2d), and kidney (Figure 2e) (P<0.01), respectively. U17Δ*iucD* showed significantly reduced CI in the heart, with a mean 4.2-fold decrease compared to U17 (Figure 2a) (P<0.05), but demonstrated no significant differences in all the other organs (P>0.05). In co-infection assays using the triple mutants, the Δ*chuT*Δ*iroD*Δ*iucD* mutants in E058 and U17 were both significantly more attenuated than each of the single mutants, with average decreases of 147-fold and 196-fold in organs tested (Figure 2) (P<0.01), respectively.

### Bactericidal effect of specific-pathogen-free (SPF) chicken serum on E058 and U17 and isogenic mutants

The ability of the isogenic mutants defective in iron acquisition systems to survive in SPF chicken serum was not affected, as tested by bactericidal assay, indicating that the iron acquisition systems may be unrelated to serum complement resistance.

### Growth of iron acquisition mutants in iron-rich and iron-restricted medium

All mutants were grown in LB with or without 200 μM 2,2'-dipyridyl (DIP). Growth patterns of the mutants were similar to those of the parent strains in both iron-rich and iron-restricted medium (data not shown).

### Bacterial invasion and intracellular viability

Analysis of the capability of mutants to enter avian macrophages was carried out using an invasion assay in the avian macrophage HD-11 cell line. Results showed no significant differences between mutant strains and the parent strains E058 and U17, with the invasion ratios varying from 0.24–0.26 (P>0.05).

To determine whether the iron uptake systems are required for intracellular survival, we compared the CFU of the wild-types and isogenic mutants recovered at 2, 4, 6, 12, and 24 hours post infection (h.p.i.). We observed similar intracellular bacterial proliferation rates, with rates of 62–65% at 2 h.p.i., which then decreased to a rate of approximately 50% at 4 h.p.i.. Rates fell sharply to approximately 10% at 6 h.p.i.. The numbers of recovered CFU at 12 and 24 h.p.i. were below detectable levels. Since iron acquisition systems are assumed to be functionally redundant, this may permit intracellular survival in the absence of one or several systems. Further, there may be TonB-independent transport systems that could compensate for the mutations in the intracellular environment.

### Histopathological lesions caused by iron acquisition defective mutants in chickens

**Figure 3:**
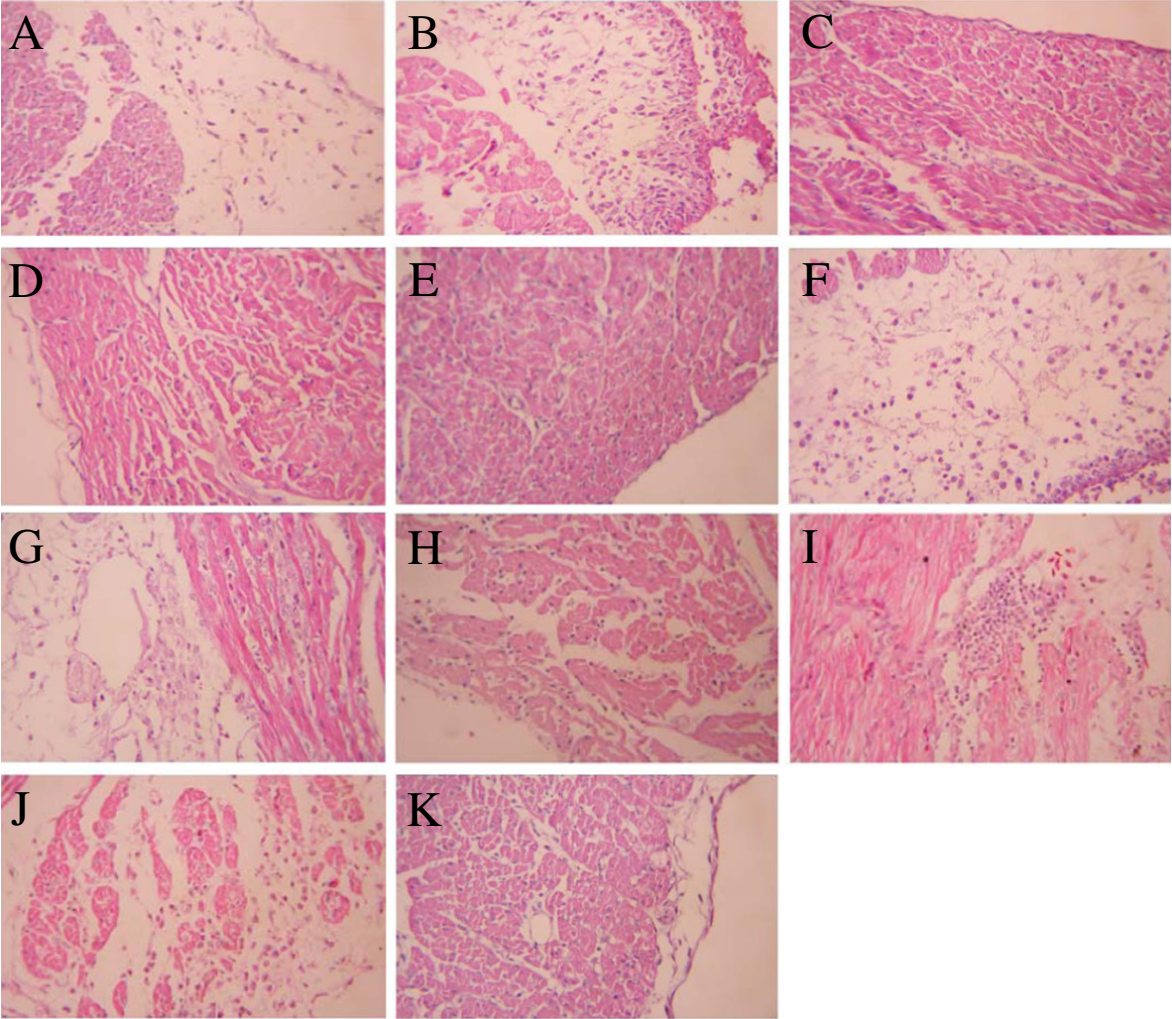
**Heart sections of chickens infected via air sac inoculation with virulent wild-type strains or iron acquisition mutants.** Magnification,×400. Heart sections of chickens infected with E058 (**A**), E058**Δ***chuT* (**B**), E058**Δ***iroD* (**C**), E058**Δ***iucD* (**D**), E058**Δ***chuT***Δ***iroD***Δ***iucD* (**E**), U17 (**F**), U17**Δ***chuT* (**G**), U17**Δ***iroD* (**H**), U17**Δ***iucD* (**I**), U17**Δ***chuT***Δ***iroD***Δ***iucD* (**J**). Heart section of a mock bird (**K**).

**Figure 4:**
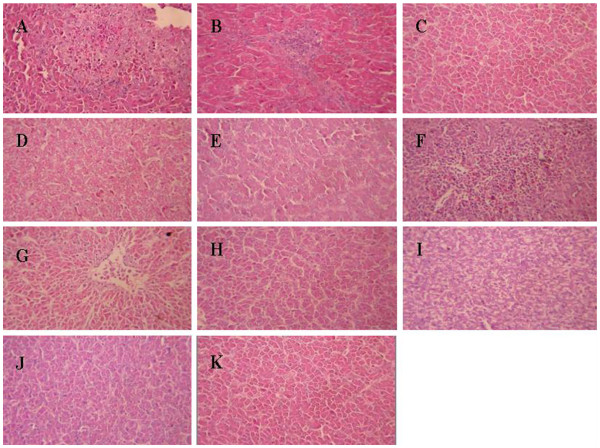
**Liver sections of chickens infected via air sac inoculation with virulent wild-type strains or iron acquisition mutants.** Magnification,×400. Liver sections of chickens infected with E058 (**A**), E058**Δ***chuT* (**B**), E058**Δ***iroD* (**C**), E058**Δ***iucD* (**D**), E058**Δ***chuT***Δ***iroD***Δ***iucD* (**E**), U17 (**F**), U17**Δ***chuT* (**G**), U17**Δ***iroD* (**H**), U17**Δ***iucD* (**I**), U17**Δ***chuT***Δ***iroD***Δ***iucD* (**J**). Liver section of a mock bird (**K**).

Histopathological lesions in chickens challenged with virulent wild-type strains or iron acquisition defective mutants were compared. The lesions in the tested organs were graded according to the lesion severity and character (Table 1). The pathological characteristics of the tested visceral organs from chickens challenged with wild-type strains were as follows. In the heart sections, unequal-sized focal necrotic lesions were present in the disintegrated muscle fibers, and fibrous exudates appeared in the epicardium (Figure 3A and Figure 3F). The liver sections showed that inflammatory cell infiltrations were present in the hepatic lobule, and numerous small fat granule vacuoles were observed in the cytoplasm (Figure 4A and Figure 4F). The lung sections revealed numerous inflammatory exudates in the bronchial cavity (data not show). However, no obvious pathological lesions were observed in the heart or liver sections of birds challenged with any of the mutant strains, except for the **Δ***chuT* mutants (Figure 3 and Figure 4). The Δ*chuT* mutants caused lesions in both the heart and liver of the challenged birds that were equivalent to the wild-type strains. This was in accordance with the results obtained in chicken colonization and persistence assays, from which the *chuT* mutation did not affect the virulence of the wild-type strains (Figure 1).

**Table 1:** Distribution and severity of histological lesions in heart, liver and lung stained with HE at 24 h post-infection in chickens challenged with wild-type strains and isogenic mutants

**Strain**	**Heart**	**Liver**	**Lung**
E058	+++	+++	+++
E058**Δ***chuT*	+++	++	+++
E058**Δ***iroD*	-	-	-
E058**Δ***iucD*	-	+	++
E058**Δ***chuT***Δ***iroD***Δ***iucD*	-	-	-
U17	+++	+++	+++
U17**Δ***chuT*	+++	+++	+++
U17**Δ***iroD*	-	-	-
U17**Δ***iucD*	+	+	++
U17**Δ***chuT***Δ***iroD***Δ***iucD*	-	-	-

HE, hematoxylin and eosin.

-, no lesions; +, mild lesions; ++, moderate lesions; +++, severe lesions.

## Discussion

APEC and UPEC are the two main subsets of ExPEC bacteria, causing diseases outside the gastrointestinal tract. Previous studies have investigated the similarities of APEC and UPEC strains by determining serogroups, virulence genotypes, and assignments to phylogenetic groups [27-30]. It has been proposed that poultry may be a candidate vehicle for *E. coli* capable of causing human urinary tract disease, based on the possible transmission of avian *E. coli* from poultry to humans, and similarities between APEC and UPEC [31-34]. Interestingly, the human UPEC isolate CFT073 was shown to be virulent in an avian respiratory infection model, but APEC isolates have not yet been found to cause disease in humans [35]. Although previous studies have been devoted to the contribution of iron uptake systems to pathogenesis of APEC or UPEC individually, the contribution of these systems to the virulence of APEC and UPEC has not been clarified simultaneously in a chicken challenge model. In this study, the roles of heme, salmochelin and aerobactin systems in the virulence of APEC E058 and UPEC U17 were assessed. Results indicated that the contribution of these three distinct iron acquisition systems to APEC E058 pathogenesis was quite similar to that of UPEC U17 when assessed simultaneously in chickens. Drawing conclusions from this study, ChuT-mediated heme transport system was generally redundant both in APEC E058 and UPEC U17 colonization and histopathological lesion formation in chickens. The IucD- mediated aerobactin synthsis played an important role in the pathogenesis of both E058 and U17, while the IroD-dependent salmochelin system provided a more critical contribution to the virulence of APEC E058 and UPEC U17.

Heme is the most abundant iron source *in vivo*, and can be utilized by certain bacterial pathogens. Hagan and Mobley demonstrated that both ChuA and Hma contribute to CFT073 heme utilization, while a ChuA heme receptor mutant was outcompeted by an Hma receptor mutant in a murine model of UTI, indicating that the ChuA receptor contributes more to heme uptake *in vivo* than does Hma [36]. Bonacorsi *et al* have presented evidence in support of the role of the *chu* heme transport system in the virulence of extraintestinal *E. coli* strains [37]. However, our results showed that ChuT contributed to a lesser extent to the virulence of APEC E058 and UPEC U17 in chickens, which implies that the heme internalized in the periplasm may still be transported by other periplasmic binding proteins or by the Hma heme transport system, which suppresses the effect of the ChuT-mediated heme transport defect.

Previous research showed that deletion of the *iroA* locus in APEC strain χ7122 resulted in decreased virulence in chickens [38]. Recent studies associated with *iro* are mainly focused on the IroN salmochelin receptor [16,39-42], while the roles of other *iro*-containing genes in *E. coli* virulence are seldom reported. IroD demonstrated higher affinity for Fe^3+^-loaded siderophores, and efficiently processed cyclic salmochelins and enterobactins into trimers, dimers, and monomers, favoring its role in cytoplasmic release of iron [21]. In this study, *iroD* was chosen to assert the role of salmochelin for ExPEC virulence. Chicken pathogenicity assay results showed that deletion of *iroD* in E058 and U17 led to highly attenuated strains of the respective wild-type strains, implying that the Iro iron uptake system plays a critical role in virulence of APEC E058 and UPEC U17 in chickens. This is in agreement with previous studies by Caza *et al*., showing that the IroD hydrolase appeared to play a predominant role in virulence of APEC compared to the IroE hydrolase [43].

When compared to commensal strains, aerobactin biosynthetic genes are more frequently detected in *E. coli* pathogenic strains, and their incidence correlates with highly pathogenic strains [44-46]. Moreover, compared to the wild-type strain, the virulence of an APEC strain deficient in aerobactin synthesis and uptake is reduced in a chicken systemic infection model [38]. Similar research showed that both salmochelin and aerobactin appeared to play a significant role in APEC virulence [38,47]. In our study, both E058**Δ***iucD* and U17**Δ***iucD* showed significantly decreased colonization compared to wild-type strains in several organs in the single-strain challenge model. This suggests that IucD-mediated aerobactin synthesis plays an important role in pathogenesis of APEC and UPEC. However, in the co-infection model, the bacterial loads of the **Δ***iucD* mutants in E058 and U17 were similar to those of the wild-type strains (P>0.05). Similarly, an **Δ***iucB***Δ***entD* double mutant, defective in synthesis of both siderophores, was rescued by co-infection with a wild-type strain in the mouse UTI model, suggesting that the exogenous siderophores synthesized by the wild-type strain are sufficient to suppress the effect of the siderophore synthesis mutations [48]. In addition, our results showed that the triple mutant **Δ***chuT***Δ***iroD***Δ***iucD* of E058 or U17 was more attenuated in the chicken challenge model than each of the single mutants, which further proved that the iron acquisition systems play important roles in the pathogenesis of APEC and UPEC in chickens. Complementation of the triple mutants by the native *iroD* gene reinstated the colonization ability of the mutant strains in most of the tested organs, confirming that the pathogenesis defect in the triple mutants is mainly mediated by the salmochelin system.

## Conclusions

Taken together, the data presented here demonstrates that both salmochelin and aerobactin systems appear to play an important role in APEC E058 and UPEC U17 virulence, while salmochelin contributed more to the virulence. The ChuT-mediated heme transport system appeared to be redundant. While no differences were observed between the mutants and their wild-type parents in other phenotypic traits tested, suggesting that other virulence mechanisms compensate for the effect of the mutations.

## Methods

### Bacterial strains, plasmids, media and culture conditions

Strains and plasmids used in this study are listed in Table 2. Bacteria were routinely cultured in Luria Bertani (LB) broth at 37°C with aeration. Antibiotics were added at the following concentrations: zeocin (Zeo), 25 μg/ml; kanamycin (Kan), 50 μg/ml; chloramphenicol (Cam), 30 μg/ml and ampicillin (Amp), 60 μg/ml.

**Table 2:** Bacterial strains and plasmids used in this study

**Strain or plasmid**	**Description**	**Reference or source**
**Strains**		
E058	APEC O2 (Ent^+^ Sal^+^ Aer^+^ Ybt^+^)	[49]
U17	UPEC nontypable (Ent^+^ Sal^+^ Aer^+^Ybt^+^), Nal^r^ Tc^r^	[5]
E058**Δ***chuT*	**Δ***chuT***::**kan, Kan^r^	This study
E058**Δ***iroD*	**Δ***iroD***::**cam, Cam^r^	This study
E058**Δ***iucD*	**Δ***iucD***::**zeo, Zeo^r^	This study
E058**Δ***chuT***Δ***iroD***Δ***iucD*	**Δ***chuT***::**kan **Δ***iroD***::**cam **Δ***iucD***::**zeo, Kan^r^ Cam^r^ Zeo^r^	This study
U17**Δ***chuT*	**Δ***chuT***::**kan, Kan^r^	This study
U17**Δ***iroD*	**Δ***iroD***::**cam, Cam^r^	This study
U17**Δ***iucD*	**Δ***iucD***::**zeo, Zeo^r^	This study
U17**Δ***chuT***Δ***iroD***Δ***iucD*	**Δ***chuT***::**kan **Δ***iroD***::**cam **Δ***iucD***::**zeo, Kan^r^ Cam^r^ Zeo^r^	This study
ReE058Trip*iroD*	pGEX-6p-1-*iroD* complementation to E058**Δ***chuT***Δ***iroD***Δ***iucD*,	This study
	Amp^r^	
ReU17Trip*iroD*	pGEX-6p-1-*iroD* complementation to U17**Δ***chuT***Δ***iroD***Δ***iucD*,	This study
	Amp^r^	
**Plasmid**		
pMD18-T Simple Vector	TA Cloning Vector	Takara
pMD-*chuT*	*chuT* cloned into pMD 18-T Simple Vector	This study
pMD-*iroD*	*iroD* cloned into pMD 18-T Simple Vector	This study
pMD-*iucD*	*iucD* cloned into pMD 18-T Simple Vector	This study
pEM7/Zeo	Zeocin-resistant cassette	Invitrogen
pUC4K	Kanamycin-resistant cassette	Invitrogen
pKD3	λ red template vector; Cam^r^ Amp^r^	[50]
pKD46	Red recombinase helper plasmid, temp sensitive; Amp^r^	[50]
pMD-*chuT*-Kan	Kan -resistant gene inserted into pMD-*chuT*	This study
pMD-*iroD*-Cam	Cam-resistant gene inserted into pMD-*iroD*	This study
pMD-*iucD*-Zeo	Zeo-resistant gene inserted into pMD-*iucD*	This study
pGEX-6p-1	expression vector	Amersham
pGEX-6p-1-*iroD*	*Bam*HI-*Eco*RI*iroD* fragment cloned into pGEX-6p-1	This study

^a^*Ent*, enterobactin; Sal, salmochelin; *Aer*, aerobactin; *Ybt*, yersiniabactin; *Kan*, kanamycin; *Cam*, chloramphenicol; *Amp*, ampicillin; *Zeo*, zeocin; *Nal*, nalidixic acid; *Tc*, tetracycline.

### Mutant construction and cloning

The **Δ***chuT*, **Δ***iroD*, and **Δ***iucD* mutants were generated in APEC E058 and UPEC U17 by allelic exchange. To enhance the numbers of recombinants, E058 and U17 were initially electroporated with pKD46 to express Red recombinase [50]. The genes were PCR amplified as described below and cloned into pMD18-T simple vector according to manufacturer’s instructions. The antibiotic resistance cassette was then inserted into the target gene. Each of the resultant constructs was then introduced into E058 or U17 by electroporation. All mutants were confirmed by PCR and verified by sequence analysis.

The **Δ***chuT* mutants, E058**Δ***chuT* and U17**Δ***chuT*, were constructed as follows: the *chuT* gene was amplified by PCR using the primers 5′-CTCGGATCCAGGATCATCACCAGGCCGTT-3′ and 5′-CTCAAGCTTTCAACGGTGATAATGCGCTG-3′. The products were cloned into pMD18-T simple vector to form pMD-*chuT*. To insert the kanamycin cassette into *chuT*, reverse PCR was adopted. The reverse PCR product was amplified from pMD-*chuT* using the primers 5′-CTCGAATTCGGTAATTACGCTATCCGG-3′ and 5′-CTCGAATTCCGTTACAGGTTCCTGAAC-3′. The kanamycin cassette was then introduced into the *chuT* genes at the *Eco*RI site.

The **Δ***iroD* E058 and U17 mutants were constructed by amplifying and cloning the fragment into pMD18-T simple vector using the primers 5′-CTCGGATCCACCATGCGTAATCGTGAC-3′ and 5′-CTCAAGCTTTACTGACTGACTTCTGGCGCGA-3′. The *cam* cassette was introduced into the *iroD* genes at the internal *Eco*RV site.

The aerobactin synthesis (*iucD*) mutants, E058**Δ***iucD* and U17**Δ***iucD*, were constructed by amplifying and cloning the *iucD* gene using the primers 5′- TCAGTCGACTCAGCATTGCTGCGTTGT-3′ and 5′-CGCGAATTCTACGT GCAGATCTCCATG −3′. The reverse PCR products were amplified from pMD-*iucD* using the primers 5′-GACGATATCTCATATGCTTCACACAGG-3′ and 5′-CCTGCATG CCTGGAGGAAGATATTCGC−3′. The *zeo* cassette was introduced into the *iucD* genes at the *Eco*RV and *Sph*I sites.

To construct the triple knockout mutant, the **Δ***iroD***Δ***iucD* double mutant was initially constructed by electroporating the disrupted *iroD* genes into the E058**Δ***iucD* and U17**Δ***iucD* competent cells. The disrupted *chuT* gene was then electroporated into the E058**Δ***iroD***Δ***iucD* and U17**Δ***iroD***Δ***iucD* double mutant competent cells to form triple mutants E058**Δ***chuT***Δ***iroD***Δ***iucD* and U17**Δ***chuT***Δ***iroD***Δ***iucD*.

### Complementation of the triple mutants using native *iroD*

For complementation analysis, the native *iroD* gene was amplified using primers 5′-CTCGGATCCATGCTGAACATGCAACAA −3′and 5′-CTCGAATTCTCAACCCTGTAGTAAACC-3′ from E058 and U17. To determine whether the sequences were in-frame, the pGEM®-T Easy vector with the *iroD* insert was sequenced by Sangon Co. (Shanghai, China). The *iroD* PCR products and expression vector pGEX-6p-1 were then digested with restriction enzymes *Bam*HI and *Eco*RI for 2 h at 37°C, and then ligated using T4 DNA ligase overnight at 4°C. Five microliters of the ligation mix were then transformed into *E. coli* DH5α and plated on LB agar containing ampicillin. Colonies were tested for the presence of *iroD* by PCR. The modified plasmid pGEX-6p-1 with the *iroD* insert was isolated from transformed DH5α and electroporated into E058**Δ***chuT***Δ***iroD***Δ***iucD* and U17**Δ***chuT***Δ***iroD***Δ***iucD* to complement the deleted *iroD* gene. The complementation strains were designated ReE058Trip*iroD* and ReU17Trip*iroD*, respectively.

### Experimental infection of chickens via the air sac

Chickens were maintained in specific-pathogen-free conditions and all experiments were conducted under the Regulations for the Administration of Affairs Concerning Experimental Animals (Approved by the State Council on October 31, 1988). Two different infection models, a single-strain challenge model and a competitive co-infection model, were used to investigate the contribution of different iron acquisition systems to the virulence of APEC and UPEC. For the single-strain challenge model, 5-week-old SPF chickens (White Leghorn, Jinan SPAFAS Poultry Co., Jinan, China) were inoculated in the left thoracic air sac with 10^8^ CFU of the wild-type strains or isogenic mutant derivatives. At 24 h post-inoculation, chickens were euthanized and examined for macroscopic lesions. The spleen, heart, anterior lobe of the liver, lung, and kidney were aseptically collected, weighed, and homogenized. Bacterial loads were determined by plating serial dilutions of the homogenates on selective LB agar medium.

For the co-infection studies, cultures of mutants and wild-type strains were mixed in a ratio of 1:1. The 5-week-old SPF chickens were inoculated with 2 × 10^8^ CFU of the mixture (1 × 10^8^ CFU for each strain, final volume of 0.5 ml) into the left thoracic air sac. Chickens were euthanized at 24 h post-infection and their spleen, heart, liver, lung, and kidney were collected, weighed, and homogenized. Serial dilutions of samples were plated on LB medium with and without appropriate antibiotics for selection of mutants or total bacteria, respectively. Then the results were showed as the log_10_ competitive index (CI). The CI was calculated for each mutant by dividing the output ratio (mutant/wild-type) by the input ratio (mutant/wild-type).

### Bactericidal assay using SPF chicken serum

All mutants were tested for their resistance to serum. Complement-sufficient SPF chicken serum was prepared and pooled from ten SPF chickens. A bactericidal assay was performed in a 96-well plate as described previously but with the following modifications [51]. SPF chicken serum was diluted to 0.5, 2.5, 5, 12.5, and 25% in pH 7.2 phosphate-buffered saline (PBS). Bacteria (10 μl containing 10^6^ CFU) were inoculated into reaction wells containing 190 μl of the diluted SPF chicken serum, 25% heat-inactivated SPF chicken serum, or PBS alone, and then incubated at 37°C for 30 min. Serial dilutions (1:10) of each well were plated onto LB agar plates. The resulting colonies were counted after 24 h incubation.

### Growth in iron-rich and iron-restricted medium

Growth of all strains in iron-rich and iron-restricted medium was examined as previously described [52]. APEC E058 and UPEC U17 and their isogenic mutants were cultured overnight in LB broth. Cultures were washed once in PBS and standardized to an optical density at 600 nm (OD_600_) of 1.0, and approximately 10^6^ CFU was inoculated into 5 ml LB with or without 200 μM 2,2'-dipyridyl (DIP). Bacterial growth was measured every hour by spectrophotometry (OD_600_). The experiment was performed in triplicate.

### Invasion assay

For invasion assays, avian macrophage cell line HD-11 was grown in Dulbecco’s modified Eagle medium (DMEM, Gibco, NY, USA) with 10% fetal bovine serum (FBS, PAA, Pasching, Australia) in 24-well cell culture plates. Cells were maintained at 37°C in a 5% CO_2_ environment and plates contained ~2 × 10^5^ cells per well. Plates were incubated for 24 h prior to invasion assay. Bacteria were inoculated onto cells with a multiplicity of infection (MOI) of 100 in cell culture medium. Inoculated cells were incubated at 37°C for 1 h with 5% CO_2_ to allow the bacteria to invade the cells. Following incubation, the medium was washed with PBS. Extracellular bacteria were then eliminated by incubation in DMEM medium containing gentamicin (100 μg/ml) at 37°C for 1.5 h. Monolayers were then washed using PBS, and the intracellular bacteria released with 1 ml 0.1% Triton X-100. One hundred microliter aliquots of the cellular suspension was inoculated into 900 μl PBS. Serial dilutions (1:10) of each well were plated onto LB agar plates. The resulting colonies were counted after 24 h of incubation. Wells containing only HD-11 were used as negative controls. The assay was performed in triplicate. The invasion ratio was determined by dividing the number of invaded bacteria by initial inoculation bacterial number.

### Intracellular survival assay

To quantify the number of viable internalized bacteria, HD-11 cells were plated and infected as described for the invasion assay. After 1 h of infection, cells were washed three times with PBS and re-incubated with cell culture medium containing 10 μg/ml of gentamicin for a further 2, 4, 6, 12, or 24 h. At each time point, cells were washed three times with PBS and lysed with 0.1% Triton X-100 for 10 min at 37°C, diluted in PBS, and plated on LB agar plates for CFU determination. The experiment was carried out in triplicate for each strain. The proliferation rate was determined by dividing the number of proliferated bacteria at each time point by initial invasion bacterial number.

### Histopathology

Three chickens were chosen from every group of the single-strain challenge model inoculated with the mutants or the wild-type strains. The sections of heart, liver, and lung were fixed in 13% neutral buffered formalin. Paraffin-embedded sections were cut at 5 μm, stained with hematoxylin and eosin, and examined for histological lesions under a 400× microscope.

### Statistical analysis

Between groups were analyzed using the Statistical Package for the Social Sciences (SPSS version 15.0, SPSS, Chicago, IL, USA). P values less than 0.05 were considered to be significant.

## Authors’ contribution

QQG carried out the mutagenesis assays, participated in the sequence alignment, and drafted the manuscript. XBW carried out the histopathological examination. HQX carried out the invasion and intracellular survival assays. YYX participated in the sequence alignment. JLL participated in the statistical analysis. DBZ participated in the chicken infection assays. SG conceived and designed the study. XFL gave an instruction in this study. All authors read and approved the final manuscript.
